# On the Use of Starchy Food for Infants

**Published:** 1869-09

**Authors:** 


### Miscellaneous.

### On the Use of Starchy Food for Infants.

At a meeting of the Obstetrical Society of London, July 7th, (*Medical Times and Gazette,*) a paper was read by Dr. Selby Norton, on Teething.

In this paper the author advocated the opinion that the maladies usually attributed to teething are due to the wide-spread and unphysiological practice of feeding infants on starch foods. He showed that starch was non-digestible by the infant stomach, partly because no minute division of the starch granules could be effected in the infant's mouth, and partly because, from the mode of feeding, the greater part, at all events, of the starch is passed at once into the infant's stomach without being rendered soluble by the ptyalin of the saliva. The diseases usually ascribed to teething—diarrhœa, convulsions and bronchitis—in the author's experience never occurred in a naturally fed child; and, on the other hand, they occurred sometimes in the first month, where the teeth obviously could exercise no baneful influence, and they occurred, too, when the gums were quite cool and natural. After considering these diseases at some length, and showing how often they could be directly traced to the irritation of bowel produced by starch food, he concluded by condemning altogether farinaceous food for infants, and advocating the sole use of cow's milk diluted with water.

Dr. T. Ballard said he was pleased to see some one come forward to support the "heretical" doctrine that teething was not a cause of infants' disease — a doctrine he had advocated many years ago. While so far, however, agreeing with the paper just read, he could not coincide in his view that starch was such a patent cause of disorder. He did not think starch, *per se*, was harmful, though of course it was not a substance on which an infant could be reared. With respect to the general subject of infant mortality, he thought that practical good would result from the inquiry if the Society could agree upon some formula of dietary for general recommendation of a simple and intelligible character. He would also lay much stress not only upon the importance of sufficient food, but on the importance of not allowing the bowels to act more than twice in the twenty-four hours. This could be effected by attention to the mode of giving the food; by not allowing an infant to suck without obtaining the food it craves, or to suck too hard to obtain it. In either case the bowels became disturbed and diarrhœa was the result. Should this occur while the child is at the breast, the too frequent motions indicate the necessity of some supplementary feeding; or, if the infant be fed entirely from the bottle, there is probably some defect in its construction or action. Where maternal milk, in sufficient quantity, could be obtained, of course no other food was requisite. Next to this came the milk of some other animal, and, where circumstances required it, to this might be added some preparation of wheaten flour.

Dr. Phillips considered it injudicious to give any farinaceous food to an infant under six months old. The practice was as physiologically incorrect as it was practically found to be hurtful. The paper read had not convinced him that no evils were ever caused by teething; but he quite believed that the evil effects ascribed to teething were often caused or increased by improper feeding. At the Children's Hospital, instructions "How to bring up Babies," had been distributed with the best effect.

Dr. Brunton said that he also objected *in toto* to giving a child farinaceous food up to six or eight months. Up to that age, where suckling could not be carried out, he gave cow's milk and water, sweetened, increasing the proportion of milk as the child grew older.

Dr. Routh said that on no point was there more evidence than against the use of starch for infants before they had teeth. For—1. The assimilation of starch depended on its conversion into sugar by the saliva, but infants secreted no saliva for the first two or three months; 2. In infants dying after the use of starchy food, examination showed that it passed through the alimentary canal unchanged; 3. The alimentary canal of a baby was that of a carnivorous animal; 4. The food supplied to purely herbivorous animals recently born was animal. *Ergo*, starchy food should not be given to infants until, at all events, the appearance of teeth. He could not agree with the recommendation of cow's milk diluted with water, as a good food for infants. The milk, before it was purchased, was generally watered, deficient in cream, acid, and wanting in sugar of milk. If used at all, it must be mixed with lime-water, and sugar of milk added in proportion of half to one ounce of lime-water, and a spoonful of sugar of milk to every half-pint of milk, with one-third water. It should be begun early, even from birth, in all cases where it was clear beforehand that the mother could not nurse long. The idea that it was wrong to mix two milks was fallacious, and his experience had proved to him that the earlier it was begun the more readily the child's stomach bore it, and in nine cases out of ten a child so prepared could be weaned readily and with safety. To one other point only would he refer—the congregation of infants in nurseries. This was a most dangerous practice. The atmosphere generated under these conditions was most baneful, probably from the quantity of ammonia generated from the urine, as well as sulphuretted hydrogen and other noxious gases from the stools. Children required air, and pure air especially. Their respiration was more rapid than adults. Such congregation of infants, was always, therefore, a great cause of infant mortality. Malignant thrush, *muguet*, and contagious diseases spread like fire in such atmosphere.—*Phila. Reporter.*

